# Is the most really the best: a review for the most selective SSRI concept three decades later

**DOI:** 10.1192/j.eurpsy.2023.899

**Published:** 2023-07-19

**Authors:** M. A. Allam

**Affiliations:** Adult psychiatry, Mental Health services - Australia, Brisbane, Australia

## Abstract

**Introduction:**

Pharmaceutical slogans presuming a particular antidepressant molecule being the best solely based on a core concept could be proved “not accurate” especially following patients’ actual exposure to the antidepressant for longer than the usual six or twelve weeks’ trials

**Objectives:**

Reviewing the current situations of **SSRI induced anhedonia** recognition and its management. Distinguishing anhedonia as a core symptoms of depression from SSRI induced anhedonia and the combination of both.

**Methods:**

Review of literatures including theses related to the same topic

**Results:**

Research suggests that, SSRIs might be more effective at treating some symptoms than others. More specifically, it has been suggested that, SSRIs might be more effective at improving symptoms such as low mood and anxiety but not anhedonia *(Argyropoulos et al. psych.pharmaology, 2013; 27(10), 869-877).* It has been proposed that, catecholaminergic antidepressants might be more effective treatments for anhedonia and emotional blunting in MDD than SSRIs *(McCabe et al. Biological psychiatry, 2010; 67(5), 439–445.* The primary effect of SSRIs is reduced processing of negative stimuli rather than increased positive stimuli. Emotional blunting is related to SSRI dose and possibly serotonergic effects on the frontal lobes and/or serotonergic modulation of midbrain dopaminergic systems projecting to the prefrontal cortex (PFC). By enhancing serotonergic transmission, SSRIs can activate the inhibitory Gamma Aminobutyric Acid (GABA) interneurons, thereby dampening the noradrenergic and dopaminergic input *( Blier. Int J Neuropsychopharmacol., 2014; 17:997–1008).*

Management of SSRI induced anhedonia includes lowering the current SSRI dose. Adding non SSRI antidepressant to the current SSRI dose or to a lowered SSRI dose. Gradual discontinuation of the SSRI and switching to another antidepressant with a different profile (SNRI) that might improve the patient’s emotional response *(Koenigs. Behav Brain Res., 2009;201:239–43).*

Bupropion is an antidepressant with less possibility to give rise to **emotional blunting.** (Tomoko et al. Neuroscience Letters., 2021; 749, 135716. agomelatine(Thome et al. Journal of neural transmission., 2015; 122(1), 3-7.Vortioxetine and others (Bing et al. frontiers in psychiatry., Jan, 2019; 10-17. are of inrerest in this regard.

**Image 2:**

**Figure:**
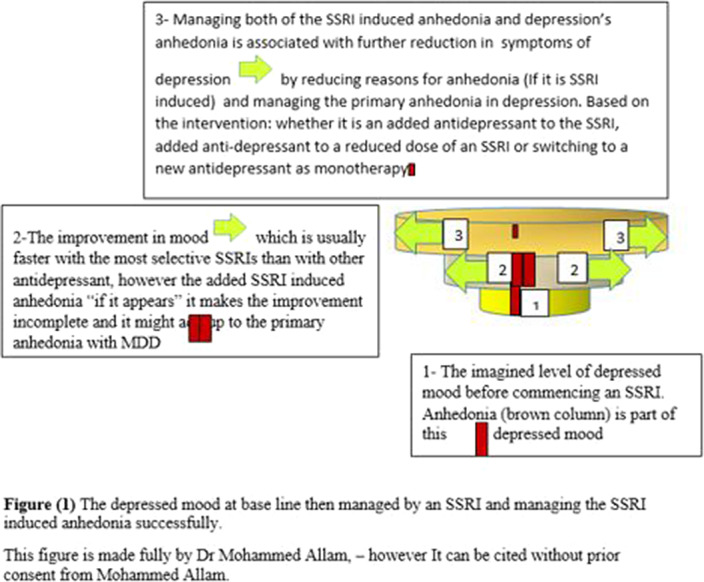

**Conclusions:**

The **most selective SSRI** concept assumes that most selective means less affinity to other receptors or secondary binding sites which might suggest less side effects and perhaps being the most efficacious. Not only serotonin but multiple neurotransmitters are in action at the downstream part of a cascade of events underpinning the etiology of MDD. MDD has heterogeneous etiology and this explains why patients respond differently. **SSRI induced anhedonia** could be tackled and we need to explore how many patients would benefit from that now and have not yet.

**Disclosure of Interest:**

None Declared

